# Down‐regulated lncRNA SLC25A5‐AS1 facilitates cell growth and inhibits apoptosis via miR‐19a‐3p/PTEN/PI3K/AKT signalling pathway in gastric cancer

**DOI:** 10.1111/jcmm.14200

**Published:** 2019-02-22

**Authors:** Xiwen Li, Xin Yan, Feng Wang, Qian Yang, Xi Luo, Jun Kong, Shaoqing Ju

**Affiliations:** ^1^ Laboratory Medicine Center Affiliated Hospital of Nantong University Nantong China; ^2^ Department of Clinical Laboratory Traditional Chinese Medicine Hospital Kunshan China; ^3^ Research Center of Clinical Medicine Affiliated Hospital of Nantong University Nantong China

**Keywords:** gastric cancer, lncRNA SLC25A5‐AS1, miR‐19a‐3p, PI3K/AKT signalling pathway, PTEN

## Abstract

Mounting evidence has illustrated the vital roles of long non‐coding RNAs (lncRNAs in gastric cancer (GC). Nevertheless, the majority of their roles and mechanisms in GC are still largely unknown. In this study, we investigate the roles of lncRNA SLC25A5‐AS1 on tumourigenesis and explore its potential mechanisms in GC. The results showed that the expressions of SLC25A5‐AS1 in GC were significantly lower than that of adjacent normal tissues, which were significantly associated with tumour size, TNM stage and lymph node metastasis. Moreover, SLC25A5‐AS1 could inhibit GC cell proliferation, induce G1/G1 cell cycle arrest and cell apoptosis in vitro, as well as GC growth in vivo. Dual‐luciferase reporter assay confirmed the direct interaction between SLC25A5‐AS1 and miR‐19a‐3p, rescue experiment showed that co‐transfection miR‐19a‐3p mimics and pcDNA‐SLC25A5‐AS1 could partially restore the ability of GC cell proliferation and the inhibition of cell apoptosis. The mechanism analyses further found that SLC25A5‐AS1 might act as a competing endogenous RNAs (ceRNA), which was involved in the derepression of PTEN expression, a target gene of miR‐19a‐3p, and regulate malignant phenotype via PI3K/AKT signalling pathway in GC. Taken together, this study indicated that SLC25A5‐AS1 was down‐regulated in GC and functioned as a suppressor in the progression of GC. Moreover, it could act as a ceRNA to regulate cellular behaviours via miR‐19a‐3p/PTEN/PI3K/AKT signalling pathway. Thus, SLC25A5‐AS1 might be served as a potential target for cancer therapeutics in GC.

## 1 INTRODUCTION

Gastric cancer (GC) is one of the most common types of digestive system tumours, which has been steadily decreasing worldwide recently. But it is still the most important leading cause of cancer mortality in the East Asia.^1^ Most patients with GC are in Chinese eastern cities, and the morbidity of GC is twice as common in women as in men.^2^ In China, the incidence of GC is the third highest among malignant tumours in males and the fourth in females.^3^ In Japan, benefit from early diagnosis, the survival rate for GC is as high as 52%, while 20%‐25% in China.^4^ The earlier the GC is identified, the better the prospect of a cure, but because of lack of identifying novel diagnostic biomarkers, the majority of GC patients is diagnosed at a late stage.^5^ Therefore, it is critical to find novel diagnostic biomarkers and effective therapeutics for GC patients.

Long non‐coding RNAs (lncRNAs) are a class of RNA transcripts which are longer than 200 nucleotides in length and lack of protein‐coding ability.^6^, ^7^ Generally, they have fewer exons than mRNA and lack extended open reading frames (ORF).^8^ LncRNAs can participate in a series of biological processes, including genomic imprinting, chromosome modification, transcription activation, cell cycle regulation, splicing translation and epigenetic regulation, etc.^9^ Since HOTAIR was discovered, humans had been increasingly concerned about the lncRNAs that was initially thought to be ‘Genomic Junk’." Increasing evidence has revealed that dysregulated expression of lncRNAs played critical roles in various disease pathogenesis, including proliferation, invasion, migration and prognosis of malignant tumours, including GC.^10^ However, the dysregulated lncRNAs and their function mechanisms in GC have not been extensively studied.

In order to reveal the potential tumourigenesis‐related lncRNAs in the progression of GC, we used microarray analysis to explore dysregulated lncRNAs in GC and found that NR_028443.1 (SLC25A5‐AS1), as a novel lncRNA, had not been reported previously. In this study, we mainly aimed to investigate the expression pattern and functions of lncRNA SLC25A5‐AS1 in GC and explore the possible mechanisms of SLC25A5‐AS1 in GC. We reported a lower expression of SLC25A5‐AS1 in GC, and several biological experiments were performed to verify SLC25A5‐AS1 could suppress GC cell proliferation, cell cycle progression and promote apoptosis by acting as competing endogenous RNAs (ceRNA) in interaction with miR‐19a‐3p through PTEN/PI3K/AKT signalling pathway.

## 2 METHODS AND MATERIALS

### 2.1 Patients and tissue samples

A total of 56 GC tissues and their pair‐matched adjacent normal tissues were obtained from patients who were diagnosed with GC at the Affiliated Hospital of Nantong University from January 2015 to May 2017. The written informed consents were obtained from all the subjects and this study was approved by the Ethics Committee of Affiliated Hospital of Nantong University (Nantong, Jiangsu, China). No patients had received chemotherapy or pre‐operative radiotherapy. All the samples were immediately infiltrated in l mL RNA latter (Qiagen, Germany) after surgical resection and stored at −80°C until for RNA isolation. The clinical parameters of GC patients in this study were presented in Table 1.

**Table 1:** The relationship between SLC25A5‐AS1 expression levels in cancer tissues and clinicopathological factors of patients with gastric cancer

Characteristics	n	*P*
Gender		0.9734
Male	35	
Female	21	
Age (y)		0.2153
≥60	29	
<60	27	
Size (cm)		**<0.01**
≥5	26	
<5	30	
Histological differentiation		0.2903
Well + Middle	8	
Poor	48	
TNM stage		**0.0202**
I + II	21	
III+IV	35	
Lymph node metastasis		**0.0353**
Yes	40	
No	16	

The bold treatment indicated a significant result featured by *P* < 0.05.

### 2.2 Cell culture

The human normal gastric epithelial cell line (GES‐1) and human GC cell lines (AGS, SGC‐7901, BGC‐823, and HGC‐27) were purchased from the Cell Resources Center of the Chinese Academy of Science. Cells were cultured in the RPMI1640 (Corning, USA) complete medium and incubated at 37°C in a humidified incubator with 5% CO_2_. The composition of the complete medium is RPMI1640 medium added with 10% foetal bovine serum (Gibco, NY, USA).

### 2.3 Microarray analysis

The Agilent Human lncRNA Microrrays V5 (4*180K, design ID: 076500) were used to analyse lncRNA expression profiles in eight samples (four GC tissues and four paired corresponding non‐tumourous tissues). Total RNA was quantified by the NanoDrop ND‐2000 (Thermo Scientific) and the RNA integrity was assessed using Agilent Bioanalyzer 2100 (Agilent Technologies). Briefly, total RNA was transcribed to double strand cDNA, then synthesized into cRNA and labelled with Cyanine‐3‐CTP. The labelled cRNAs were hybridized onto the microarray. After washing, the arrays were scanned by the Agilent Scanner G2505C (Agilent Technologies). GeneSpring (version 13.1, Agilent Technologies) was employed to analyse the raw data. Differentially expressed genes or lncRNAs were then identified through fold change as well as *P*value calculated with *t*‐test. The threshold set for up‐ and down‐regulated lncRNAs was a fold change 2.0 and *P* ≤ 0.05. Finally, hierarchical clustering was performed to display the distinguishable genes' expression pattern among samples.

### 2.4 RNA extraction and quantitative real‐time polymerase chain reaction (qRT‐PCR)

Total RNA was extracted using RNA extraction Kit (Thermo Fisher Scientific, Waltham, MA, USA). qRT‐PCR assays were performed by Light Cycler® 480 SYBR Mix (Roche, Germany) in a total volume of 20 µL on LightCycler® 480 real‐time PCR system. The expression levels of lncRNA, miRNA or mRNA was normalized to the expression of 18S rRNA or U6 using the 2^–ΔΔct^ method. Primers used for amplifying specific genes were purchased from GenePharma (Shanghai, China) and the sequences were as follows, SLC25A5‐AS1, forward: 5′‐ACGGAAC TTGTGATTACACTAT‐3′, reverse: 5′‐CCTTCACTGGGTAAGCATT‐3′; PTEN, forward: 5′‐ACACGACGGGAAGACAAGTT‐3′, reverse: 5′‐TCCTCTGGTCCTGG TATGAAG‐3′. 18S rRNA, forward: 5′‐GTAACCCGTTGAACCCCATT‐3′, reverse: 5′‐CCATCCAATCGGTAGTAGCG‐3′; miR‐19a‐3p, forward: 5′‐ACACTCCAGCTG GGTGTGCAAATCTATGCAA‐3′, reverse: 5′‐CTCAACTGGTGTCGTGGAGTCGG CAATTCAGTTGAGTCAGTTTT‐3′; U6, forward: 5′‐AGAGCCTGTGGTGTCCG‐3′, reverse: 5′‐CATCTTCAAAGCACTTCCCT‐3′.

### 2.5 Cell transfection, plasmid construction and cell nucleus/cytoplasm fraction isolation

GC cells were incubated in six‐well plates until 80% confluence, then the pcDNA3.1 and shRNA vectors were transfected by Lipofectamine 3000 (Thermo Fisher Scientific, USA) in serum‐free medium. After 4‐6 hours of incubation, cell culture media was changed into the RPMI1640 medium and was added with 10% foetal calf serum. After the other 48 hours of incubation, cell lysates were harvested for qRT‐PCR and Western blot analysis. Plasmid pcDNA3.1+ SLC25A5‐AS1, pcDNA3.1+ vector and short hairpin RNA (shRNA) sequences were synthesized by GenePharma Corporation (Suzhou, China), The target sequences of shRNA SLC25A5‐AS1 are as follows: shRNA1: 5′‐GCCAGTGAAACCAGACGAAAT‐3′, shRNA2: 5′‐GCAACTGCAGCT GAACCTTAT‐3′, shRNA3: 5′‐GGTAAAGTGCCCTTGGATTGA‐3′, shRNA4: 5′‐ GGTTGTACCCAGAAGGTTAAG‐3′. For cell nucleus/cytoplasm fraction isolation, Cytoplasmic & Nuclear RNA Purification Kit (Norgen Biotek, Canada) was used to separate cell nucleus and cell cytoplasm. The RNAs were collected for qRT‐PCR analyses respectively.

### 2.6 Proliferation assay

Cell proliferation was measured by Cell Counting Kit‐8 (CCK‐8) and colony formation assays. 5 × 10^3^ per well of GC cells were seeded into a 96‐well plate after transfection. Then 10 μL of CCK‐8 (Dojindo, Kumamoto, Japan) was added into each well at 1, 2, 3 and 4 days. After 2 hours of incubation, the absorbance value was measured at 450 nm using a Microplate Reader. In regard to colony forming assay, cells were seeded in six‐well plates at a concentration of 5 × 10^2^ per well and incubated in complete medium and incubated at 37°C in a incubator with 5% CO_2_ for 14 days, then the cells were fixed with methanol and stained using 0.1% crystal violet.

### 2.7 Analysis of apoptosis and cell cycle progression

The cells were collected and washed with cold PBS after 48 hours of transfection. According to the manufacturer's protocol, cells for apoptotis were analysed using flow cytometric with 7AAD and PE, FITC Apoptosis Detection Kits (BD Biosciences, USA) and cells for cycle distribution were fixed with 70% ethanol overnight at 4°C, then washed with cold PBS and incubated with 100 μL RNase A for 30 minutes at 37°C, then performed with flow cytometric after stained with PE.

### 2.8 Luciferase reporter assays

Cells were seeded at a concentration of 5×10^4^ cells/well in 24‐well plates and co‐transfected with pmirGLO‐SLC25A5‐AS1‐WT, pmirGLO‐ SLC25A5‐AS1‐MUT reporter plasmids and mimics NC, miR‐19a‐3p mimics accordingly. After 48 hours, cells were lysed using passive lysis buffer and the luciferase activity was measured by GloMax 20/20 Luminometer using the Dual‐Luciferase Reporter Assay System following manufacturer's protocol.

### 2.9 Western blot analysis

Total protein was extracted from GC cells after transfection for 48 hours using Radio‐Immunoprecipitation Assay (RIPA) protein extraction reagent (Beyotime, Beijing, China). Protein lysates were mixed with loading buffer and separated based on their molecular weight on SDS/PAGE gels, then transferred onto a Polyvinylidene Fluoride (PVDF, Millipore) membrane. The membrane was blocked with fat‐free milk (5%) for 2 hours at room temperature and incubated with first antibody at 4°C overnight. Then removed the first antibody with cold TBST three times and incubated with the secondary antibody for 2 hours at room temperature. The first antibody included BCL‐2, BAX, p‐AKT, AKT, p‐PI3K, PI3K, P27, cyclinD1 (1:1000 dilution) (Cell Signalling Technologies, Danvers, MA, USA). The bound antibodies were detected using Chemiluminescence imaging system (BIO‐RAD, USA) with GAPDH used as a control.

### 2.10 Tumour formation in nude mice

Animal experiments were approved by the Institutional Animal Care and Use Committee of the Affiliated Hospital of Nantong University. The SGC7901 cells were stably transfected with pcDNA SLC25A5‐AS1 or empty vector. Nude mice (BALB/c Nude, 4 weeks old) were purchased from the Experimental Animal Centre of Nantong University, all of which were subcutaneously injected into the ventral side with 10^7^ cells of each mouse for tumour formation assays. The ventral tumour volume was measured at 10, 15, 20, 25 and 30 days. Tumour models were gathered and stained with immunohistochemistry.

### 2.11 Immunohistochemistry

The tumours were fixed in 4% paraformaldehyde solution at 4°C for 48 hours, then embedded in paraffin and sectioned into 4 μm thick. Slides were deparaffinized through a series of xylene and graded alcohols. For antigen retrieval, slides were heated in citrate buffer (pH 6.0) in sub‐boiling temperature for 10 minutes. Then endogenous peroxidase activity was blocked by 3% H_2_O_2_ for 15 minutes, and then the slides were incubated with primary antibody overnight at 4°C. Next day, the secondary antibody (Gene Tech, Shanghai, China) was applied for 30 minutes at room temperature. Following Diaminobenzidine (DAB) staining for appropriate time, haematoxylin staining was performed. The antibodies against Ki‐67 (1:1000) were purchased from CST (USA).

### 2.12 Statistical analysis

Graphad Prism 6 was used for the statistical analysis. The significance of differences between groups was estimated by Student's *t*‐test and multiple groups were estimated with one‐way ANOVA. Spearman's correlation analyses were analysed between SLC25A5‐AS1and PTEN or miR‐19a‐3p expression levels in GC tissues. *P* < 0.05 was considered statistically significant. Each experiment was conducted in triplicate.

## 3 RESULTS

### 3.1 SLC25A5‐AS1 was down‐regulated in GC tissues and associated with tumour progression

**Figure 1:**
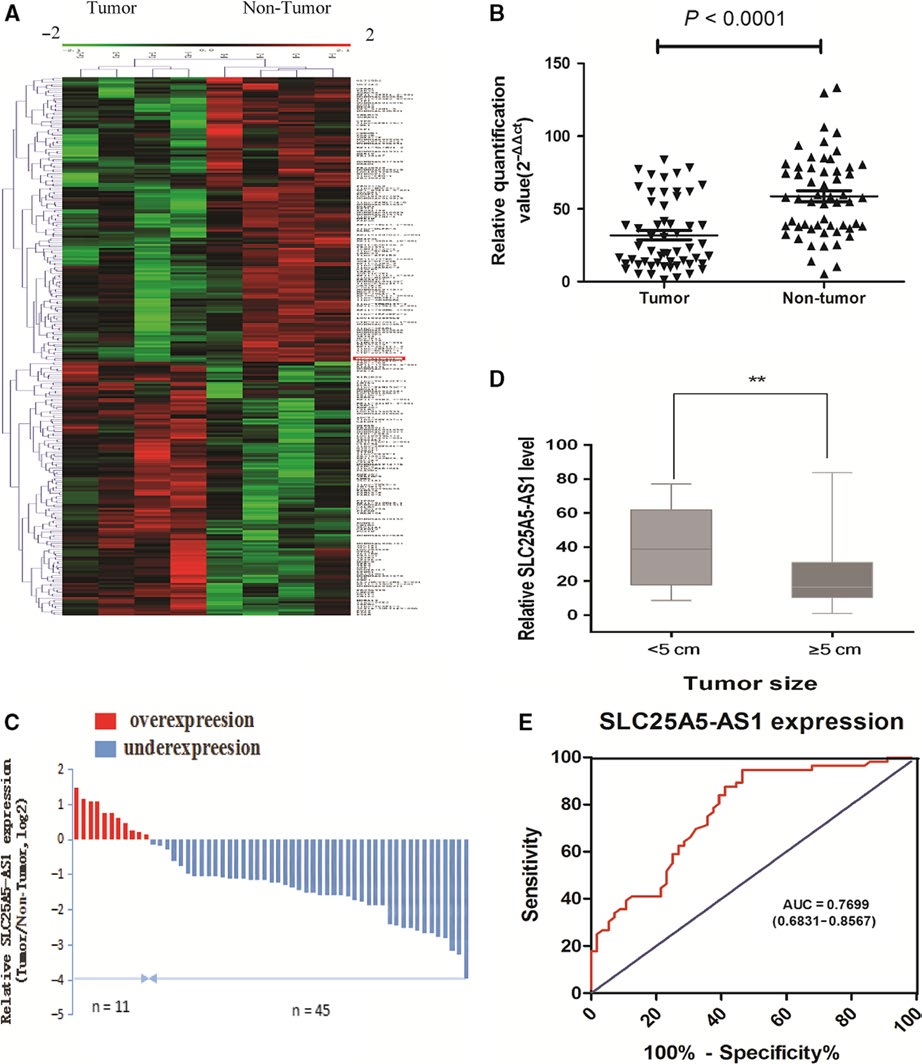
Expression of SLC25A5‐AS1 in GC tissues and its association with clinicopathological characteristics. A, Hierarchical clustering analysis of the lncRNAs that were differentially expressed (fold change >2 or <−2, *P* < 0.05) between GC tissues (Tumour) and paired non‐tumour tissues (Non‐Tumour). B, SLC25A5‐AS1 was detected in 56 pairs of GC tissues by qRT‐PCR. The level of SLC25A5‐AS1 was significantly decreased in GC tissues, as compared with matched adjacent normal tissues, *P* < 0.0001. C, SLC25A5‐AS1 expression was significantly down‐regulated in 80.4% (45 of 56 paired). The results were normalized to adjacent normal tissues and shown as log2 (2^–ΔΔCt^). D, SLC25A5‐AS1 expression was significantly decreased in patients with advanced tumour size, **P < 0.01. E, ROC curve for prediction of GC based on SLC25A5‐AS1 expression level, the AUC was 0.7699 (95% CI 0.6831‐0.8567)

Firstly, lncRNA microarray was used to analyse abnormal expressed lncRNAs in four paired GC tissues and corresponding adjacent normal tissues. We identified 87 up‐regulated lncRNAs and 117 down‐regulated lncRNAs that were filtered by the criteria of fold change >2 and *P* < 0.05 between cancer and adjacent normal specimens, hierarchical clustering analysis showed a clear distinction on most significantly dysregulated expression of lncRNAs in cancer as depicted in the heat map (Figure 1A). Among them, we selected an anti‐sense lncRNA, named SLC25A5‐AS1, which was down‐regulated in GC. In order to further assess whether SLC25A5‐AS1 was dysregulated in GC, we performed qRT‐PCR to analyse the expression of SLC25A5‐AS1 in 56 GC tissues. We found that the level of SLC25A5‐AS1 was significantly decreased in GC tissues, as compared with matched adjacent normal tissues, *P* < 0.0001 (Figure 1B). SLC25A5‐AS1 expression was down‐regulated in 80.4% (45 of 56 paired) GC tissues (Figure 1C). Furthermore, we investigated whether its expression was associated with the patients’ clinicopathological characteristics. As shown in Figure 1D, SLC25A5‐AS1 level was associated with tumour size (*P* < 0.01), and lymph node metastasis (*P* < 0.05) and TNM stage as well (*P* < 0.05, Table 1), but not associated with sex, ages or histological differentiation, all *P* > 0.05. Additionally, the receiver operating characteristic (ROC) curve was constructed to assess diagnostic value of SLC25A5‐AS1 level. The results showed that the area under the ROC curve of SLC25A5‐AS1 was 0.7699 (95% CI 0.6831‐0.8567), indicating SLC25A5‐ AS1 might be a good diagnostic marker for GC (Figure 1E). These data suggested that SLC25A5‐AS1 might play an important role in tumour development and metastasis in GC.

### 3.2 SLC25A5‐AS1 inhibits cell proliferation, cell cycle progression and promotes apoptosis in GC cells in vitro

**Figure 2:**
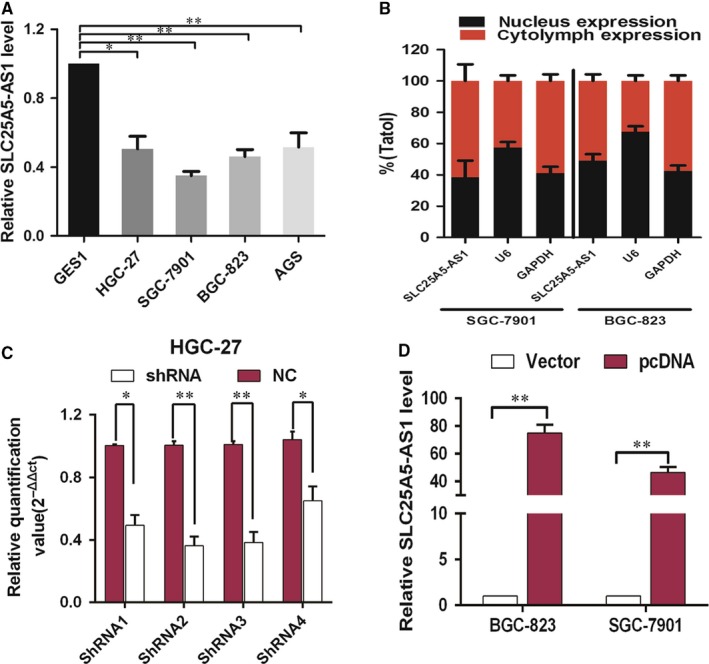
Expression of SLC25A5‐AS1 in GC cell lines and subcellar location. A, qRT‐PCR analyses of SLC25A5‐AS1 expression in GC cell lines (HGC‐27, BGC‐823, SGC‐7901 and AGS) and normal gastric mucosa epithelial cells (GES1). B, Relative nuclear and cytoplasmic levels of SLC25A5‐AS1 in GC cells assessed by qRT‐PCR with U6 and GAPDH as nuclear and cytoplasmic markers respectively. (C and D) Relative SLC25A5‐AS1 expression level in shRNA‐treated HGC‐27 cells, and pcDNA‐SLC25A5‐AS1‐treated BGC‐823 and SGC‐7901 cells. *P < 0.05, **P < 0.01

**Figure 3:**
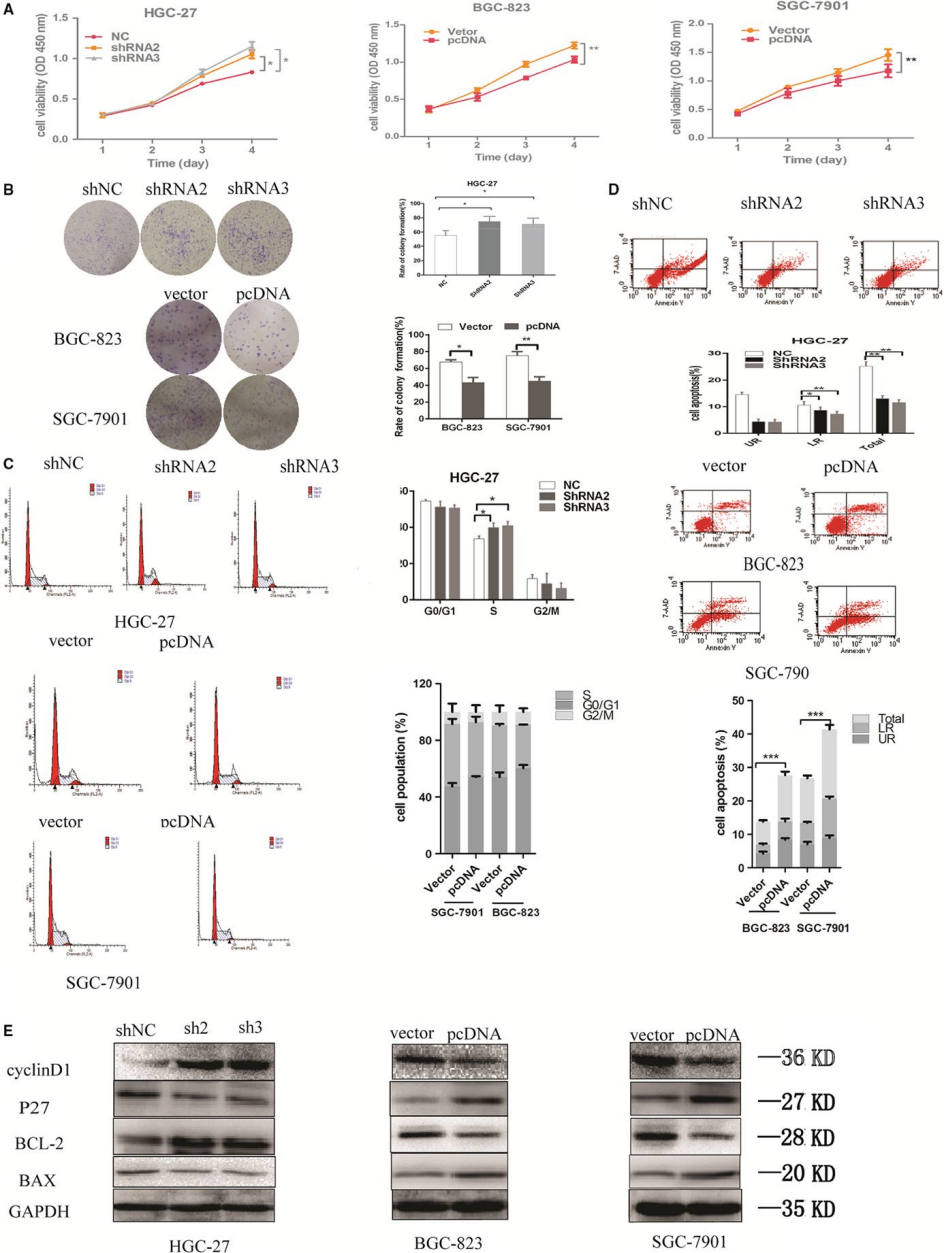
SLC25A5‐AS1 regulates GC cell proliferation and apoptosis in vitro. (A and B) CCK‐8 assays and colony formation assays were used to determine cell proliferation of HGC‐27, BGC‐823 and SGC‐7901 cells after transfection of shRNA and pcDNA of SLC25A5‐AS1. (C and D) Flow cytometry analyses of cell cycle and cell apoptosis distribution in HGC‐27, BGC‐823 and SGC‐7901 cells after transfection with shRNA and pcDNA of SLC25A5‐AS1 (E) Western blotting assays for the expression of cycle‐associated and apoptosis‐related proteins in HGC‐27, BGC‐823 and SGC‐7901cells after transfection with shRNA and pcDNA of SLC25A5‐AS1. *P < 0.05, **P < 0.01, ***P < 0.001

We next further examined the expression levels of SLC25A5‐AS1 in GES1 (a normal gastric epithelial cell line) and four human GC cells (SGC‐7901, BGC‐823, AGS and HGC‐27). As compared with the GES1, the level of SLC25A5‐AS1 was significantly decreased in four GC cells (Figure 2A). Herein, we chose SGC‐7901, BGC‐823 and HGC‐27 cells for further investigation. Meanwhile, qRT‐PCR analyses, nuclear and cytoplasmic fractions suggested that SLC25A5‐AS1 was mainly located in the cytolymph (Figure 2B). To assess the biological functions of SLC25A5‐AS1 in GC cells, we performed loss‐ or gain‐of function experiments. We firstly constructed four RNA vectors, named shRNA1, shRNA2, shRNA3 and shRNA4, shNC as a control, and overexpressed vector, named pcDNA, empty vector as a control. The transfection efficiency was performed by qRT‐PCR and the results showed that SLC25A5‐AS1 expression was significantly reduced in HGC‐27 cells after transfection with shRNA, especially shRNA2, shRNA3, and significantly increased in SGC‐7901 and BGC‐823 cells after transfection with pcDNA (Figure 2C,D). Then, CCK‐8 assays and colony formation analyses demonstrated that the ability of cell proliferation was significantly increased in shRNA2 and shRAN3 transfected HGC‐27 cells than that of shNC group, on the other hand, the ability of cell proliferation was significantly decreased in pcDNA transfected BGC‐823 and SGC‐7901 cells than that of vector control group (Figure 3A,B). It is commonly known that dysregulation of cell cycle and apoptosis are crucial reasons for tumour cell proliferation. To investigate SLC25A5‐AS1 pro‐proliferation mechanism, we performed flow cytometry analyses to assess the effects of SLC25A5‐AS1 on cell cycle and apoptosis. The results revealed that overexpression of SLC25A5‐AS1 led to a conspicuous cell cycle arrest in the G0/G1 phase and a significant decrease in cells in S‐phase. Conversely, the S‐phase cells were increased after knockdown of SLC25A5‐AS1 in GC cells (Figure 3C). Furthermore, the percentage of early and total apoptotic cells was significantly increased in BGC‐823 and SGC‐7901 cells after overexpressed pcDNA‐SLC25A5‐AS1 than that of vector control, whereas, the percentage of early and total apoptotic cells was significantly decreased in HGC‐27 cells after knockdown SLC25A5‐AS1 than that of shNC group (Figure 3D). In addition, we also detected cycle‐associated proteins (cyclinD1, P27) and apoptosis‐associated proteins (BCL‐2, BAX) by Western blot analysis, the results revealed that the protein level of cyclinD1 and BCL‐2 was increased while P27 and BAX was decreased in SLC25A5‐AS1‐knockdown HGC‐27 cells, while in SLC25A5‐AS1‐overexpressed BGC‐823 and SGC‐7901 cells, as expected, we obtained opposite results (Figure 3E).

### 3.3 SLC25A5‐AS1 regulates GC growth in vivo

**Figure 4:**
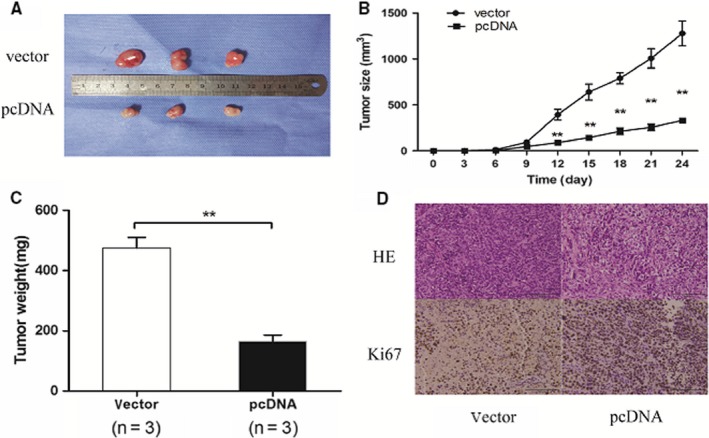
SLC25A5‐AS1 inhibits GC cell tumour growth in vivo. A, Empty vector or pCDNA‐SLC25A5‐AS1 were transfected into SGC‐7901 cells, which were injected in the nude mice (n = 3) respectively. Tumours formed in pcDNA‐SLC25A5‐ AS1 group were significantly smaller than that of empty vector group. B, Tumour volumes were measured every 3 d from 0 to 24 d after inoculation. C, Tumour weights were represented as means of tumour weights ± SD. D, The tumour sections were under H&E staining and IHC staining using antibodies against ki‐67, bar = 100 μm. **P < 0.01

According to the findings of SLC25A5‐AS1 inhibiting GC cell proliferation, cell cycle and apoptosis in vitro, we next examined the effect of SLC25A5‐AS1 on GC growth in vivo. We firstly established a nude mice model by subcutaneously injecting the SGC7901 cells into the ventral side. Two weeks after the subcutaneous injection, we found that the tumours formed in the pcDNA‐SLC25A5‐AS1 transfected group had smaller tumour size than those in vector control group (Figure 4A,B). Moreover, the tumours formed in the pcDNA‐SLC25A5‐AS1 transfected group had lighter weight than those in vector control group (Figure 4C). These results were further confirmed by immunohistochemical staining and HE staining of tumour tissues. The results indicated that lower expression of Ki‐67 was observed in pcDNA‐SLC25A5‐AS1‐ treated group (Figure 4D). Taken together, these vivo data demonstrated that SLC25A5‐AS1 might act as an anti‐oncogene which could suppress GC cell proliferation.

### 3.4 SLC25A5‐AS1 functions as a ceRNA directly interacted with miR‐19a‐3p

**Figure 5:**
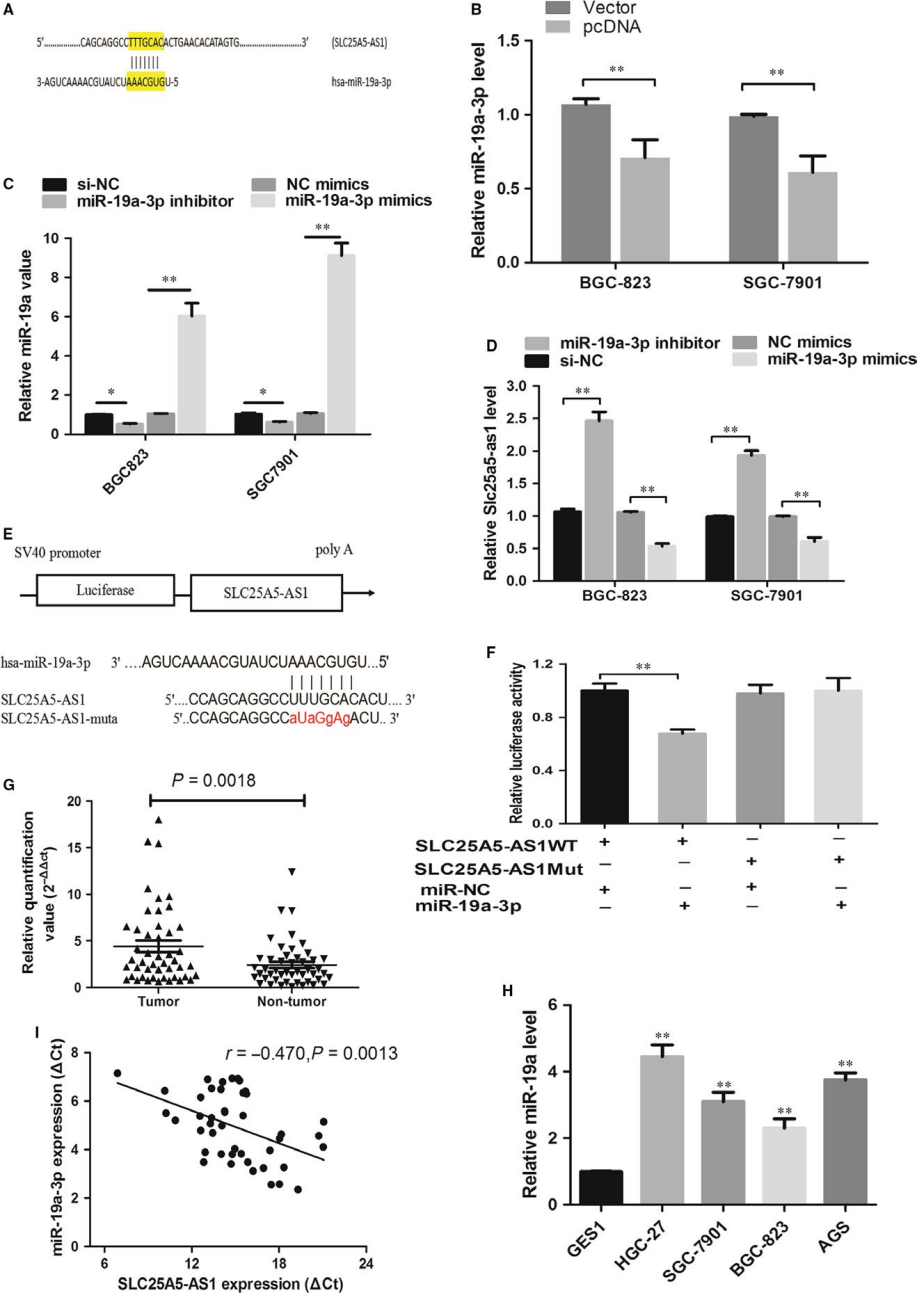
SLC25A5‐AS1 directly interacted with miR‐19a‐3p. A, Bioinformatics software predicted miR‐19a‐3p has one binding site on the SLC25A5‐AS1 transcript. B, qRT‐PCR detected miR‐19a‐3p expression after pcDNA‐SLC25A5‐AS1 transfection in BGC‐823 and SGC‐7901 cells. C, Expression of miR‐19a‐3p after transfected with miR‐19a‐3p inhibitor and mimics in BGC‐823 and SGC‐7901 cells. D, qRT‐PCR analysed SLC25A5‐AS1 expression after miR‐19a‐3p knockdown or up‐regulation in BGC‐823 and SGC‐7901 cells. E, The miR‐19a‐3p binding site predicted in the sequence of SLC25A5‐AS1, red font as mutant locus. F, Relative firefly/renilla luminescence was analysed in BGC‐823 cells co‐transfected with miR‐19a‐3p mimics and wild‐type or mutant SLC25A5‐AS1 sequence constructed luciferase plasmid. (G and H) miR‐19a‐3p expression levels were analysed in GC tissues and GC cell lines by qRT‐PCR. I, Correlation analysis was performed between SLC25A5‐AS1 expression levels and miR‐19a‐3p expression levels in GC tissues, **P < 0.01

Increasing researches have suggested that lncRNA can function as a ceRNA in regulating the biological functions of specific miRNAs in various cancers. Combined with previous studies ,we found that SLC25A5 (ANT2), a natural sense transcript of SLC25A5‐AS1, could suppress tumour growth and down‐regulate miR‐19a through the PI3K/AKT pathway in hepatocellular carcinoma cells.^11^ To determine whether SLC25A5‐AS1 could interact with miR‐19a, we found one potential binding site between miR‐19a‐3p and SLC25A5‐AS1 by miRanda (Figure 5A). The expression levels of miR‐19a‐3p were obviously down‐regulated in SLC25A5‐AS1 overexpressed SGC‐7901 and BGC‐823 cells (Figure 5B). In addition, qRT‐PCR analyses also found that transfection with miR‐19a‐3p mimics and inhibitor could enhance and decrease SLC25A5‐AS1 expression, respectively (Figure 5C,D). To further evaluate whether SLC25A5‐AS1 and miR‐19a‐3p were directly interacted, we constructed luciferase reporter targeting SLC25A5‐AS1 wild‐type sequences (Figure 5E, red font as mutant locus). Dual‐luciferase reporter gene assay showed that miR‐19a‐3p mimic reduced the luciferase activity in GC cells after transfected with the SLC25A5‐AS1 wild‐type vector, but not the mutant ones (Figure 5F). Moreover, we found that miR‐19a‐3p level was obviously increased in GC tissues and GC cells, as compared with adjacent non‐tumour tissues and GES1 cells respectively (Figure 5G,H). In addition, miR‐19a‐3p expression had a significant negative correlation with SLC25A5‐AS1 expression in GC tissues, Pearson *r* = −0.470, *P* = 0.0013 (Figure 5I). These data revealed that SLC25A5‐AS1 might directly interact with miR‐19a‐3p and act as a ceRNA.

### 3.5 Aberrant miR‐19a‐3p expression partially rescued the SLC25A5‐AS1‐ induced tumour‐suppressive effects on GC cells

**Figure 6:**
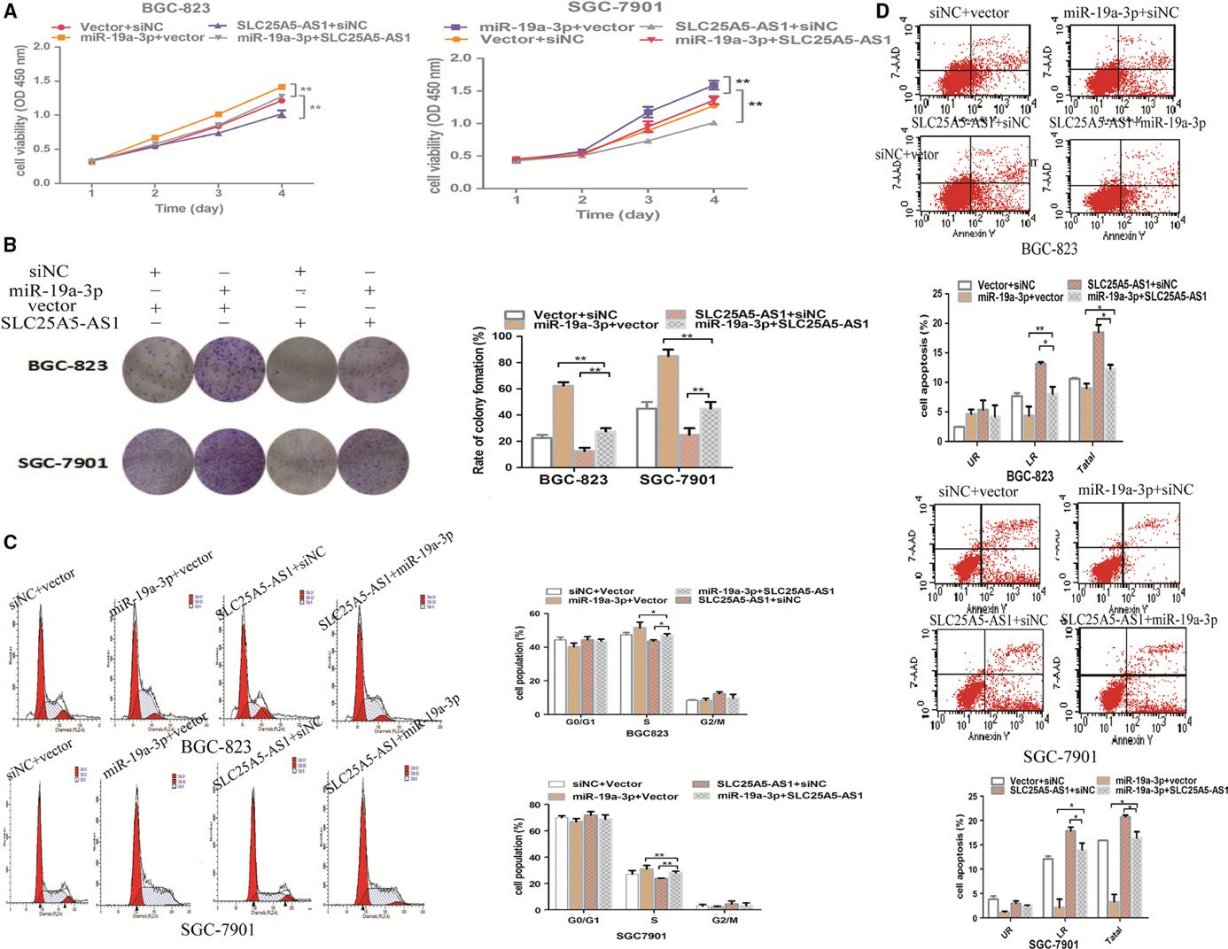
SLC25A5‐AS1 inhibits cell growth and promotes apoptosis by negatively regulation of miR‐19a‐3p. A, CCK‐8 assays were performed to analyse cell proliferation viability of each group. The 450 nm absorption was assayed after culture from 1 to 4 d. B, Colony formation assays were also used to determine the cell proliferation viability. The histogram showed the rate of colony formation of cells in different group. C, Cell cycle distribution of different group cells was measured using flow cytometry analysis. The histogram showed the proportion of cells in each phase of the cell cycle in different groups. D, After transfected for 48 h, cells were collected and stained with 7AAD and PE and then examined by flow cytometry. The histogram showed the apoptosis rate obtained from each group. *P < 0.05, **P < 0.01

Previous studies have demonstrated that miR‐19a plays critical roles in multiple malignant tumours and participates in a variety of biological processes, including cell proliferation, cell cycle and apoptosis, etc.^12^, ^13^ To further confirm that the mutual regulation between SLC25A5‐AS1 and miR‐19a‐3p on these biological processes, miR‐19a‐3p mimics were co‐transfected with SLC25A5‐AS1 in GC cells to perform rescue experiments. We divided BGC‐823 and SGC‐7901 into four groups: vector+miR‐NC, vector+miR‐19a‐3p mimics, pcDNA‐SLC25A5‐AS1+miR‐NC and pcDNA‐SLC25A5‐AS1+miR‐19a‐3p mimics. The CCK‐8 and colony formation assays, as well as cell cycle and apoptosis analyses showed that as compared with pcDNA‐SLC25A5‐AS1+ miR‐NC group, pcDNA‐SLC25A5‐AS1+miR‐19a‐3p group could reverse the reduced cell proliferation, the increased cell apoptosis and the arrest of G0/G1 cell cycle (Figure 6A‐D). Overall, these results revealed that the carcinostatic effects of SLC25A5‐AS1 were at least partly through inhibiting miR‐19a‐3p.

### 3.6 SLC25A5‐AS1 inhibits cell growth and promotes apoptosis via miR‐19a‐3p ‐PTEN/PI3K/AKT signalling pathway

**Figure 7:**
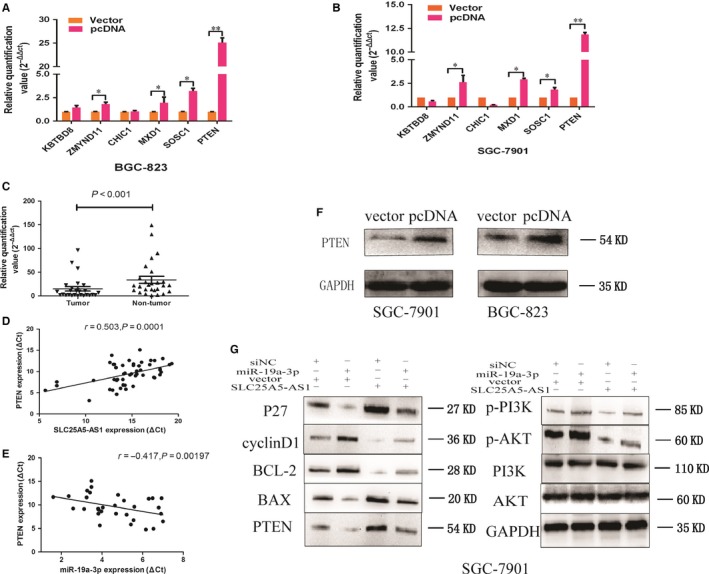
SLC25A5‐AS1 modulated the miR‐19a‐3p/PTEN/PI3K/AKT signalling pathway. (A, B) qRT‐PCR were used to detected the relative expression of potential mRNA targets, included KBTBD8, ZMYND11, CHIC1, MXD1, SOSC1 and PTEN, of miR‐19a‐3p in BGC‐823 and SGC‐7901 cells transfected with or without pcDNA‐SLC25A5‐AS1. C, MRNA expression level of PTEN in GC and normal tissues were detected by qRT‐PCR. D, The correlation analysis was performed between the expression level of SLC25A5‐AS1 and PTEN in GC tissues by qRT‐PCR. E, The correlation analysis between the expression level of miR‐19a‐3p and PTEN in GC tissues was detected by qRT‐PCR. F, After transfected for 72 h, cells were collected and total proteins were extracted for Western blot. The protein levels of PTEN in BGC‐823 and SGC‐7901 cells transfected with or without pcDNA‐SLC25A5‐AS1 were analysed by Western blot. G, Rescue experiments of Western blot analyses were used to analyse the protein levels of p27, cyclinD1, BCL‐2, BAX, PTEN, p‐PI3K, PI3K, p‐AKT and AKT in cells of each group. *P < 0.05, **P < 0.01

To find the target of miR‐19a‐3p, we used Targetscan, miRanda to predict potential target mRNAs, such as KBTBD8, ZMYND11, CHIC1, MXD1, SOSC1, PTEN. Previous studies demonstrated that miR‐19a regulated the cell proliferation and apoptosis by targeting PTEN in osteosarcoma stem cells and hepatocellular carcinoma.^12^, ^13^ Moreover, we found that the expression levels of PTEN mRNA were obviously up‐regulated when overexpressed SLC25A5‐AS1 in SGC‐7901 and BGC‐823 cells (Figure 7A,B). Moreover, the protein level of PTEN was significant increased when we overexpressed SLC25A5‐AS1 in BGC‐823 and SGC‐7901 cells (Figure 7F). Additionally, as compared with adjacent non‐tumour tissues, PTEN mRNA level was obviously decreased in GC tissues (Figure 7C). Besides, PTEN mRNA levels were positively correlated with SLC25A5‐AS1 (Pearson *r* = 0.503, *P* = 0.0001) (Figure 7D) and negatively correlated with miR‐19a‐3p (Pearson *r* = −0.417, *P* = 0.00197) (Figure 7E). Combined with previous studies and our experiments data, we confirmed that PTEN was a target of miR‐19a‐3p in GC and verified the relationship between PTEN and SLC25A5‐AS1. In addition, miR‐19a also plays critical roles through activating the PI3K/AKT pathway in GC.^14^, ^15^ Thus, we explored whether SLC25A5‐AS1 regulating cell proliferation, cell cycle and apoptosis were directly interacted with miR‐19a‐3p via the PTEN/PI3K/AKT pathway. It showed that overexpression of SLC25A5‐AS1 decreased the expression of BCL‐2 and cyclinD1, while P27 and BAX were significantly increased in GC cells. On the other hand, overexpression of miR‐19a‐3p increased the expression of BCL‐2 and cyclinD1, while P27 and BAX were significantly decreased in GC cells. Furthermore, we co‐transfected pcDNA‐SLC25A5‐AS1 and miR‐19a‐3p mimics in GC cells, and found that SLC25A5‐AS1 partially could restore the protein levels of PTEN, p‐PI3K, p‐AKT, as compared with miR‐19a‐3p mimics transfected ones, whereas, the protein levels of PI3K, AKT had no change (Figure 7G). Overall, these data indicated that an interaction with miR‐19a‐3p was necessary for SLC25A5‐AS1 to inhibit cell growth and promote apoptosis via the PTEN/PI3K/AKT signalling pathway in GC.

## 4 DISCUSSION

The development of GC is a multi‐stage and multi‐factorial process, which is one of the main fatal tumours that threaten our health in China.^3^, ^16^ For a long time, many tumour researchers have paid more attention to multiple genetic and epigenetic alterations on coding genes.^17^, ^18^ With the development of microarray and RNA sequencing technology,^19^ more and more non‐coding RNAs (ncRNAs) were characterized. According to the positional relationship between lncRNA and the protein‐coding genes, they can be divided into sense lncRNA, anti‐sense lncRNA, bidirectional lncRNA, intergenic lncRNA and intronic lncRNA.^20^, ^21^, ^22^ Increasing evidence has demonstrated that anti‐sense lncRNAs are featured as crucial regulators during tumourigenesis, and play critical roles in tumour proliferation, cell cycle and apoptosis, which maybe act as valuable diagnostic and prognostic biomarkers for cancer.^23^ Zhang et al^24^ reported that anti‐sense lncRNA FOXC2‐AS1 was up‐regulated in doxorubicin‐resistant osteosarcoma by increasing the expression of FOXC2. Miao et al^25^ reported that lncRNA FOXF1‐AS1 inhibited epithelial‐mesenchymal transition, stemness and metastasis of non‐small cell lung cancer (NSCLC) cells. He et al^26^ demonstrated that lncRNA FEZF1‐AS1 was overexpressed in NSCLC tissues compared with adjacent normal tissues and associated with lymph node metastasis, poor differentiation grade and advanced TNM stage in NSCLC patients.

Microarray and RNA‐seq are effective tools for high‐throughput detection of lncRNA expression, which can simply screen out a large number of genes associated with tumour development. To explore the differential expression of lncRNAs in GC tissues and corresponding adjacent normal tissues, we extracted the total RNA in four paired GC and corresponding adjacent normal tissues and conducted a microarray, the results were verified by qRT‐PCR. In our present study, we found that SLC25A5‐AS1, an anti‐sense lncRNA that located in X chromosome q24, was firstly identified as a dysregulated lncRNA in GC. There was less report about the genes located in X chromosome than those located in other chromosomes. XIST was the first reported tumour‐associated gene located in X chromosome.^27^ Previous study showed that XIST knockdown could inhibit GC progression and metastasis through modulating the expression of EZH2.^28^ It seems that XIST gene is up‐regulated in a variety of non‐sex‐related tumours in both humans and mice.^29^ In our present study, we found that SLC25A5‐AS1 was significantly decreased in GC tissues and cells. The relationship between SLC25A5‐AS1 expression levels and clinicopathological characteristics was further investigated. We found that SLC25A5‐AS1 expression was significantly associated with tumour size, TNM stage and lymph node metastasis. Knockdown of SLC25A5‐AS1 induced HGC27 cells proliferation, suppressed the apoptosis and promoted the G1/S transition of the cell cycle. Moreover, overexpression of SLC25A5‐AS1 repressed BGC823 and SGC7901 cell proliferation, induced apoptosis, arrested more cells in G0/G1 phase and suppressed tumour growth in vivo. These data suggested that SLC25A5‐AS1 might be a novel anti‐oncogene lncRNA which was involved in the progression of GC.

Emerging evidence revealed that lncRNAs might function as scaffolds, guides, decoys of other molecules and ceRNAs as well.^30^, ^31^, ^32^, ^33^ LncRNAs, mainly located in cytoplasm, can affect post‐transcriptional expression of gene by alternative splicing, transformation, export and translocation enhancement of messenger RNA or reducing protein translation. Besides, they can act as ceRNAsor as miRNA sponges adsorbing onto the binding sequence of miRNAs.^34^ For example, lncRNA GAS5 levels were significantly reduced in prostate cancer, and GAS5 overexpression can inhibit tumour progression in part through its inhibitory effects on the expression and activity of miR‐103.^35^ Similar reports indicated that CASC2 could function as a ceRNA to modulate miR‐181a and promote glioma growth and sensitize glioma cells to TMZ.^36^ Ma et al^37^ revealed a reciprocal repression between lncRNA GCASPC and miR‐17‐3p in gallbladder cancer. The theory of ceRNA considers that lncRNA, mRNA and pseudogene transcription products can be competitive with miRNAs, function as a ‘molecular sponge’, reduce the number of miRNAs interacting with mRNA, regulate the expression of miRNAs downstream target genes in post‐transcriptional level.^38^ In our current study, we also found that SLC25A5‐AS1 might function as a ceRNA directly interacting with miR‐19a‐3p by dual‐luciferase reporter assay. Furthermore, the effect of pcDNA‐SLC25A5‐AS1 was partially attenuated the ability of miR‐19a‐3p mimics on GC cell proliferation indicating that SLC25A5‐AS1 may interact with miR‐19a‐3p to regulate the progress of GC. Previous studies confirmed that miR‐19a could act as an oncogene in GC,^39^ lung cancer,^40^ colon cancer,^41^ breast cancer^42^ and so on. Moreover, it could regulate the PTEN/AKT signalling pathway in myeloma and^43^ breast cancer.^44^ Thus, we further explored whether SLC25A5‐AS1 could directly interact with miR‐19a‐3p via the PTEN/PI3K/AKT pathway to regulate tumour progression. Our results revealed that miR‐19a‐3p promoted cellular behaviours via PTEN/PI3K/AKT pathway and could regulate cell cycle regulator cyclinD1 and P27, apoptosis‐related BCL‐2 and BAX expression. Additionally, SLC25A5‐AS1 could inversely regulate miR‐19a‐3p expression and inhibit PTEN/AKT signalling through positively regulating the expression of PETN. Collectively, SLC25A5‐AS1 interacts with miR‐19a‐3p to inhibit cell growth and promote apoptosis via the PTEN/PI3K/AKT signalling pathway.

In conclusion, our study revealed that SLC25A5‐AS1 was down‐regulated in GC and functioned as a suppressor gene in the progression of GC. Additionally, SLC25A5‐AS1 might act as a ceRNA to sponge miR‐19a‐3p and regulate cellular behaviours via PTEN/PI3K/AKT signalling pathway in GC. Taken together, our present results indicated that SLC25A5‐AS1 might be served as a potential target for cancer therapy in GC.

## CONFLICT OF INTEREST

The authors declare that there is no conflict of interest.
