# Correction to Down‐regulated lncRNA SLC25A5‐AS1 facilitates cell growth and inhibits apoptosis via miR‐19a‐3p/PTEN/PI3K/AKT signalling pathway in gastric cancer

**DOI:** 10.1111/jcmm.17830

**Published:** 2023-09-18

In Xiwen Li et al.,^1^ the colony formation analyses for BGC‐823 (vector) and SGC‐7901 of Figure 3B are incorrect. In addition, the labels for BGC‐823 and SGC‐7901 are reverse in Figure 3E. The correct figures are shown below. The authors confirm that all results and conclusions of this article remain unchanged.

**FIGURE 3:**
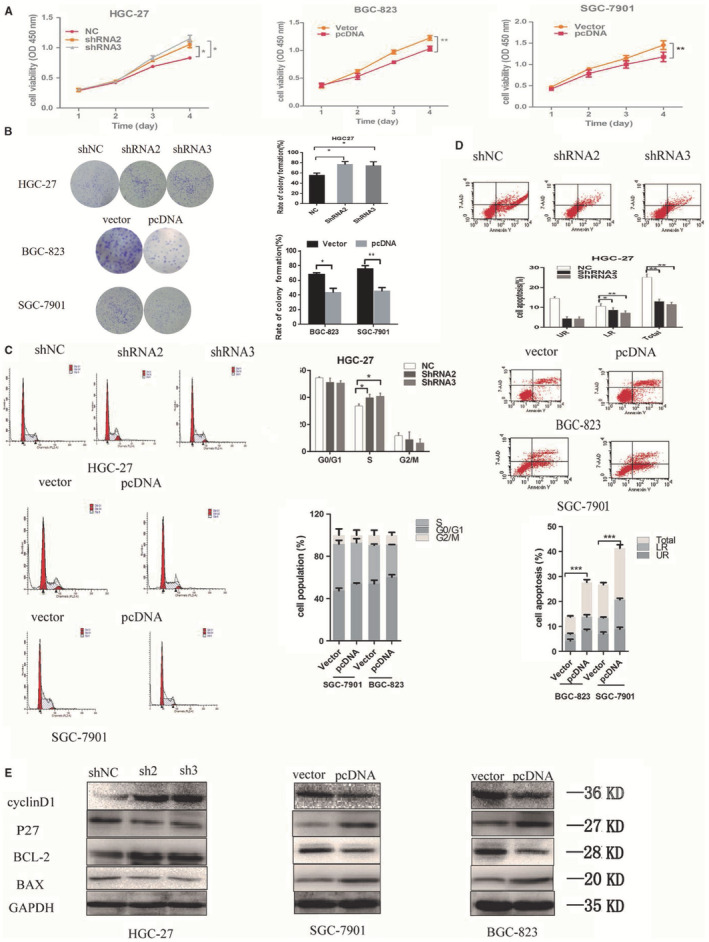
SLC25A5‐AS1 regulates GC cell proliferation and apoptosis in vitro. (A, B) CCK‐8 assays and colony formation assays were used to determine cell proliferation of HGC‐27, BGC‐823 and SGC‐7901 cells after transfection of shRNA and pcDNA of SLC25A5‐AS1. (C, D) Flow cytometry analyses of cell cycle and cell apoptosis distribution in HGC‐27, BGC‐823 and SGC‐7901 cells after transfection with shRNA and pcDNA of SLC25A5‐AS1. (E) Western blotting assays for the expression of cycle‐associated and apoptosis‐related proteins in HGC‐27, BGC‐823 and SGC‐7901 cells after transfection with shRNA and pcDNA of SLC25A5‐AS1. *p < 0.05, **p < 0.01, ***p < 0.001.
